# Effects of Baduanjin exercise on cardiac rehabilitation after percutaneous coronary intervention: a systematic review and meta-analysis of randomized controlled trials

**DOI:** 10.1186/s40001-025-03031-2

**Published:** 2025-09-29

**Authors:** Yingkun Zhao, Wujiao Wang, Yi Cai, Bo Liu, Peifen Chang, Tianli Li, Peng Yang

**Affiliations:** 1 Dongzhimen Hospital of Beijing University of Chinese Medicine, Beijing, 100700 China; 2 National Integrated Traditional and Western Medicine Center for Cardiovascular Disease, China-Japan Friendship Hospital, Beijing, 100029 China; 3 Beijing University of Chinese Medicine, Beijing, 100029 China

**Keywords:** Baduanjin, Percutaneous coronary intervention, Meta-analysis, Systematic

## Abstract

**Objectives:**

To systematically evaluate the effects of Baduanjin exercise on cardiac rehabilitation after percutaneous coronary intervention (PCI).

**Methods:**

From the time the database was constructed to May 28, 2025, Eight databases and two registry systems, including Web of Science, Cochrane Library, PubMed, Embase, China National Knowledge Infrastructure (CNKI), Wanfang database, China Science and Technology Journal Database (VIP), Chinese Biomedical Literature database (CBM), Clinical Trials, and the China Clinical Trials Registry were searched—clinical randomized controlled trials (RCTs) of Baduanjin in treating patients after PCI were retrieved. The primary outcomes were the 6-min walk test (6MWT) and left ventricular ejection fraction (LVEF). Secondary outcomes were Major adverse cardiovascular event (MACE), Seattle Angina Questionnaire (SAQ), Self-Rating Anxiety Scale (SAS), Self-Rating Depression Scale (SDS), Anaerobic threshold (AT), Metabolic equivalent of task (METs), and Maximal oxygen consumption (VO_2_ max). The quality of the included studies was assessed using the Cochrane Risk of Bias assessment tool, version 2.0 (RoB 2). Meta-analysis was performed using RevMan 5.4 software. Sensitivity analysis and subgroup analysis were performed using Stata software. In addition, Publication bias was evaluated using funnel plots and Egger's test.

**Results:**

A total of 56 RCTs involving 5152 patients were included in the study. Compared with the control group, the Baduanjin group showed superior improvement in LVEF (MD = 5.55%, 95% CI [4.28%, 6.82%], *P* < 0.01, *I*^2^ = 94%), 6MWT (MD = 57.68m, 95% CI [40.20m, 75.17m], *P* < 0.01, *I*^2^ = 100%), MACE (RR = 0.33, 95% CI [0.26, 0.42], *P* < 0.01, *I*^2^ = 0%), SAQ-PL (MD = 7.49 points, 95% CI [4.78 points, 10.20 points], *P* < 0.01, *I*^2^ = 91%), SAQ-AS (MD = 12.88 points, 95% CI [10.76 points, 15.00 points], *P* < 0.01, *I*^2^ = 77%), SAQ-DS (MD = 11.30 points, 95% CI [5.14 points, 17.45 points], *P* < 0.01, *I*^2^ = 98%), SAQ-AF (MD = 10.90 points, 95% CI [6.05 points, 15.75 points], *P* < 0.01, *I*^2^ = 98%), SAQ-TS (MD = 8.04 points, 95% CI [2.30 points, 13.78 points], *P* < 0.01, *I*^2^ = 98%), SAS (MD = − 7.01 points, 95% CI [− 8.05 points, − 5.96 points], *P* < 0.01, *I*^2^ = 58%), SDS (MD = − 6.67 points, 95% CI [− 8.34 points, − 5.00 points], *P* < 0.01, *I*^2^ = 89%), VO_2_peak (MD = 1.81 mL/kg/min, 95% CI [0.82 mL/kg/min, 2.80 mL/kg/min], *P* < 0.01, *I*^2^ = 96%), AT (MD = 1.18 mL/kg/min, 95% CI [0.66 mL/kg/min, 1.69 mL/kg/min], *P* < 0.01, *I*^2^ = 96%), and METs (MD = 0.61 METs, 95% CI [0.32 METs, 0.90 METs], *P* < 0.01, *I*^2^ = 83%) when compared to control groups. Subgroup analysis showed that patients with chronic coronary syndromes (CCS) were more suitable as a target population. Improvement in LVEF was better with intervention duration of 1–3 months, whereas improvement in 6MWT was better with intervention longer than 3 months, and it is not recommended to combine Baduanjin with aerobic exercise.

**Conclusions:**

Baduanjin can improve cardiopulmonary function, alleviate clinical symptoms, improve quality of life, adjust mental state, and reduce the incidence of MACE in patients after PCI.

**Systematic review registration:**

CRD42024626379.

**Supplementary Information:**

The online version contains supplementary material available at 10.1186/s40001-025-03031-2.

## Introduction

Percutaneous coronary intervention (PCI) is the primary approach to revascularisation in patients with obstructive coronary heart disease (CHD) [1]. The incidence of CHD is gradually increasing. A study has demonstrated that CHD accounts for 2.2% of the overall global burden of disease and 32.7% of cardiovascular disease [2]. As a result, the proportion of PCI procedures is gradually increasing. Although PCI can effectively restore blood flow to the coronary arteries, there is still a risk of compromising cardiac function [3]. PCI can cause physical injury and ischemia–reperfusion injury to cardiomyocytes, which can lead to calcium overload [4] and exacerbate structural and functional damage to cardiomyocytes. Post-operative inflammation promotes thrombosis, which further compromises myocardial perfusion. Cardiomyocyte remodeling and mitochondrial dysfunction contribute to a reduction in overall ventricular systolic and diastolic efficiency [5]. As a result, these factors can trigger arrhythmias and severely compromise the recovery of cardiac function in patients after PCI. Therefore, cardiac rehabilitation (CR) plays a crucial role in the post-PCI period.

CR aims to facilitate physiological recovery and improve patients'quality of life through multidimensional approaches, including exercise training, nutritional guidance, psychological support, and other relevant interventions [6]. CR is effective in reducing readmission rates, mortality, and the risk of cardiovascular events in patients with cardiovascular disease while improving health-related quality of life [7]. International guidelines support CR programs [8]. The American Heart Association, the European Society of Cardiology, and the Chinese Society of Cardiology all recommended CR as a Class I intervention for patients with CHD [9–11].

In recent years, Baduanjin has gained increasing attention as one of the exercise modalities for CR. Baduanjin is a traditional Chinese exercise method that combines physical activity, breathing, and psychological regulation [12]. It maximizes the physical and mental health of people with chronic conditions by emphasizing respiratory regulation, musculoskeletal relaxation, and relaxation mindfulness. To date, a growing number of clinical studies have demonstrated the benefits of the Baduanjin intervention in cardiac rehabilitation [13]. A previous study using qualitative content analysis found that Baduanjin improves cardiac function, heart rate variability, and psychological well-being in patients with ST-elevated myocardial infarction after PCI [14]. Baduanjin has also been included in the recommended aerobic exercises in the technical guidelines for cardiac rehabilitation in China [15]. Therefore, we will systematically evaluate the effectiveness of Baduanjin in facilitating cardiac rehabilitation for patients undergoing PCI, thereby providing evidence-based support for its use as an approach to cardiac rehabilitation after PCI.

## Methods

This systematic review was reported following the PRISMA 2020 statement [16]. The protocol was registered with PROSPERO (CRD42024626379).

### Search strategy

From the time the database was constructed to May 28, 2025, Eight databases and two registry systems, including Web of Science, Cochrane Library, PubMed, Embase, China National Knowledge Infrastructure (CNKI), Wanfang database, China Science and Technology Journal Database (VIP), Chinese Biomedical Literature database (CBM), Clinical Trials, and the China Clinical Trials Registry were searched—clinical randomized controlled trials (RCTs) of Baduanjin in treating patients after PCI were retrieved. The supplementary material provides detailed search strategies and results of the database searches.

### Inclusion criteria

The inclusion criteria were as follows:

1. Patients: Patients after PCI regardless of age, gender, ethnicity or nationality;
2. Intervention: Conventional treatment (CT) combined with Baduanjin.
3. Comparator: CT, which involved standard pharmacological regimens such as aspirin, statins, beta-blockers, and calcium channel blockers (CCBs) and could be combined with guideline-based rehabilitation programs when clinically indicated.
4. Outcomes: The primary outcomes were the 6-min walk test (6MWT) and left ventricular ejection fraction (LVEF). Secondary outcomes were Major adverse cardiovascular event (MACE), Seattle Angina Questionnaire (SAQ), Self-Rating Anxiety Scale (SAS), Self-Rating Depression Scale (SDS), Anaerobic threshold (AT), Metabolic equivalent of task (METs), and Maximal oxygen consumption (VO_2_ max). N-terminal pro-brain natriuretic peptide (NT-proBNP) and cardiac troponin I (cTnI) were used as safety indicators.
5. Study types: RCTs.

### Exclusion criteria

1. Non-RCTs.
2. Patients with serious comorbidities that could confound cardiac function—such as ESRD, malignancies, severe chronic liver or pulmonary diseases—as well as pregnancy and morbid obesity, were excluded.
3. The treatment group used any other traditional Chinese medicine treatments, such as acupuncture, acupressure, and herbal medicine.
4. Duplicate literature.
5. Full-text unavailable.
6. Lack of complete data.

### Data extraction

Two researchers independently screened the literature based on the retrieval strategy. Initially, duplicate articles were removed. Subsequently, studies that did not meet the inclusion and exclusion criteria were excluded based on their titles and abstracts. The remaining articles were subject to a second-round screening, where further selection was made based on the full text. The articles included by both researchers were compared, and any disagreements were resolved through consultation and discussion with a third researcher. After finalizing the included literature, information extraction was conducted using Excel, encompassing: (1) basic article information: title, first author, publishing journal, and publication date; (2) Basic study information: sample size, gender ratio, mean age, diagnostic criteria, intervention measures for both the control and experimental groups, intervention duration, follow-up time, outcome indicators, and adverse events; and (3) Risk assessment factors: methods for generating and concealing the random sequence, implementation of blinding, and records of dropout.

### Assessment of bias risk

The research quality of the included RCTs was assessed using the Cochrane Collaboration's risk of bias assessment tool, version 2.0 (RoB 2). This process included an assessment of the randomization process, deviations from the intended interventions, missing outcome data, measurement of the outcome, selection of the reported outcome, and overall risk of bias. The assessment of bias was categorized into three levels: “low risk”, “some concerns”, and “high risk”. Two researchers performed the assessments independently, and any discrepancies were resolved through discussion with a third researcher.

### Statistical analysis

A meta-analysis of outcome measures was performed using RevMan 5.4. For categorical data, the relative risk (RR) was used. For continuous data, the mean difference (MD) was used as the effect size measure if the units were the same; if the units were different, the standardized mean difference (SMD) was used. All effect sizes were presented with 95% confidence intervals (95% CI). Statistical significance was considered at *P* < 0.05 for both types of data. Heterogeneity was assessed using the *I*^2^ statistic and the *p* value. Specifically, if *I*^2^ < 50% or the *P* > 0.1, heterogeneity was considered acceptable, and a fixed effects model was applied. If *I*^2^ ≥ 50% or *P* < 0.1, heterogeneity was considered significant, and a random-effects model was applied [17]. In such cases, subgroup analysis or sensitivity analysis is recommended. If significant heterogeneity is observed, sensitivity analysis should be performed using Stata software to identify potential sources of heterogeneity by sequentially excluding studies. Subgroup analysis should then be performed on the following predefined subgroups: type of heart disease; whether the control group combined aerobic exercise; and duration of intervention. This will allow us to observe whether they affect heterogeneity and the associated *p* values. To further explore the sources of heterogeneity, a simultaneous meta-regression analysis was conducted. In addition, publication bias should be assessed using the Egger and Begg tests if the number of publications for the outcome indicator is greater than ten [18].

### Grading of the evidence

The quality of evidence was assessed using the GRADE method [19]. It was categorized as high, moderate, low, or very low. RCTs received a high initial grade by default and were downgraded according to pre-specified criteria: risk of bias, inconsistency, indirectness, imprecision, and other considerations.

## Results

### Literature search results and study characteristics

**Fig. 1:**
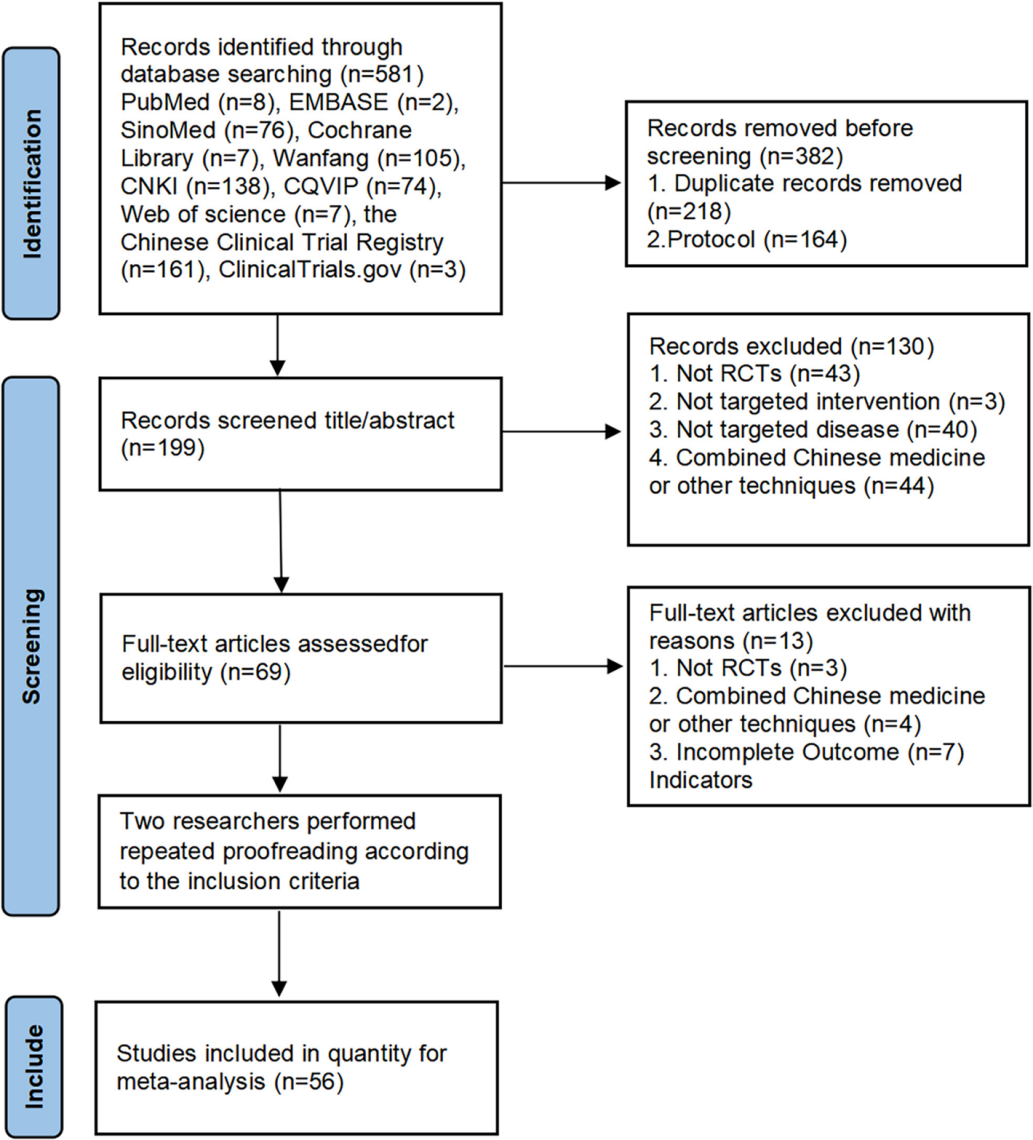
Flow diagram of the literature search

Based on the inclusion and exclusion criteria, a total of 581 studies were retrieved. 199 papers were obtained after excluding duplicates and protocols, and 69 papers were obtained after excluding non-RCT, non-Baduanjin, non-patients after PCI, and inappropriate intervention studies. Finally, 56 studies [14, 20–34, 36-74] were included (Fig. 1).

A total of 56 studies were included, there were 5152 participants, 2578 in the treatment group and 2574 in the control group. Detailed characteristics of the included studies are shown in Table 1.

**Table 1:** General characteristics of the studies

Study	Sample		Age (years)		Interventions		Classification	Treatment duration	Outcome index
	Trial	Control	Intervention	Control	Intervention	Control			
Kang, L. 2024 [35]	60	60	52.2 ± 10.9	53.0 ± 10.7	CT + BDJ	CT	AMI	6 months	②⑪
Chen, M. G. 2020 [23]	48	48	59.98 ± 10.86	61.49 ± 11.54	CT + BDJ	CT	AMI	24 months	①
Cao, X. 2024 [14]	79	80	55.32 ± 9.58	54.76 ± 9.22	CT + BDJ	CT	STEMI	1 week	②
Cai, Y. H. 2022 [21]	28	29	56.5 ± 8.05	53.83 ± 12.01	CT + BDJ	CT	AMI	3 months	①
Cai, Y. 2022 [20]	45	45	49.58 ± 9.41	49.47 ± 9.32	CT + BDJ	CT	AMI	2 weeks	②
Chai, Q. 2025 [22]	54	54	59.4 ± 10.2	60.3 ± 11.1	CT + BDJ	CT	STEMI	3 months	①③
Deng, S. C. 2022 [24]	51	51	64.56 ± 5.05	64.51 ± 5.12	CT + BDJ	CT	SAP	6 months	②③
Du, Y. C. 2023 [25]	53	53	60.98 ± 6.04	60.39 ± 6.12	CT + BDJ	CT	CHD	3 months	①②③
Fu, Y. T. 2021 [26]	50	50	50.81 ± 4.50	50.33 ± 4.98	CT + BDJ	CT	AMI	6 months	②③
Gao, M. M.2022 [27]	39	39	69.88 ± 2.47	69.48 ± 2.47	CT + BDJ	CT	SAP	3 months	①②⑥
Gong, L. 2021 [28]	30	30	68.67 ± 8.46	67.43 ± 9.22	CT + BDJ	CT	ACS	4 weeks	①④⑤⑦⑪
Gong, L. et al. 2021 [29]	30	30	62.64 ± 8.42	62.35 ± 8.21	CT + BDJ	CT	CHD	3 months	⑨
Gu, F. 2018 [30]	50	50	59.42 ± 8.99	59.32 ± 10.02	CT + BDJ	CT	CHD	3 months	⑪
He, X. 2023 [31]	30	30	65.11 ± 3.65	64.34 ± 4.12	CT + BDJ	CT	CHD	3 months	①②⑥
Hu, C. C. 2023 [32]	60	60	57.16 ± 5.28	56.84 ± 6.27	CT + BDJ	CT	ACS	3 months	①③⑥⑦⑧
Hu, L. 2018 [33]	40	40	47.70 ± 9.12	47.75 ± 9.89	CT + BDJ	CT	CHD	8 weeks	①④⑤⑪
Hua, L. 2018 [34]	60	60	NA	NA	CT + BDJ	CT	CHD	1 week	④
Kang, L. 2021 [36]	30	30	51.27 ± 10.62	51.33 ± 9.95	CT + BDJ	CT	AMI	8 weeks	①②⑥⑨⑪
Li, Y. Q. 2024 [37]	27	28	NA	57.29 ± 8.85	CT + BDJ	CT	AMI	3 months	②③
Liu, F. 2024 [38]	39	39	65.64 ± 7.05	66.05 ± 7.12	CT + BDJ	CT	AMI	3 months	①③④⑤⑦⑩
Liu, G. G. 2021 [39]	30	30	56.21 ± 10.44	57.32 ± 11.36	CT + BDJ	CT	STEMI	12 weeks	①②③⑥
Liu, L. 2023 [40]	44	43	67.24士8.28	67.31士8.34	CT + BDJ	CT	AMI	1 month	①⑨⑩
Liu, M. L. 2023 [41]	65	66	56.71 ± 10.56	55.73 ± 10.80	CT + BDJ	CT	AMI	3 months	①⑪
Liu, P. P. 2024 [42]	61	61	59.37 ± 4.94	58.72 ± 5.51	CT + BDJ	CT	STEMI	3 months	①②⑩⑧
Ni, X. S. 2021 [43]	60	60	56.51 ± 18.15	55.20 ± 15.61	CT + BDJ	CT	CHD	6 months	①②③④⑤
Qiu, J. Z. 2024 [44]	40	40	64.39 ± 7.35	62.69 ± 8.77	CT + BDJ	CT	CHD	3 months	②⑨⑩
Ren, Z. J. 2024 [45]	50	50	63.27 ± 5.27	64.95 ± 4.96	CT + BDJ	CT	AMI	6 months	①②⑥⑦
Rong, C. L. 2022 [46]	80	80	61.52 ± 3.14	61.61 ± 3.11	CT + BDJ	CT	CHD	6 months	②⑪③
Shi, N. 2021 [47]	60	60	58.25 ± 5.84	58.86 ± 6.45	CT + BDJ	CT	CHD	12 weeks	⑩
Shi, L. S. 2023 [48]	32	32	57.74 ± 11.67	60.06 ± 10.99	CT + BDJ	CT	AMI	3 months	①⑪
Sun, C. J. 2024 [49]	55	55	61.34 ± 5.48	62.17 ± 5.64	CT + BDJ	CT	AMI	3 months	①③
Tang, T. 2019 [50]	46	47	60.02 ± 8.66	61.38 ± 9.21	CT + BDJ	CT	CHD	3 months	②
Wang, J. M. 2018 [51]	75	75	59.3士15.4	58.8士12.5	CT + BDJ	CT	AMI	6 months	①②③
Wang, J. 2021 [52]	20	20	61.95 ± 9.79	62.15 ± 9.74	CT + BDJ	CT	CHD	6 months	②③
Wang, J. J. 2019 [53]	55	55	58.32 ± 9.74	60.32 ± 7.23	CT + BDJ	CT	CHD	5 months	①④⑤
Wang, J. 2024 [54]	46	46	60.02 + 6.38	59.96 + 6.39	CT + BDJ	CT	AMI	2 weeks	①
Wang,X 2023 [55]	40	40	52.0 ± 16.7	52.5 ± 15.5	CT + BDJ	CT	CHD	8 weeks	①②④⑤⑥
Wang, X. J. 2019 [56]	30	30	60.11 ± 8.54	59.51 ± 8.93	CT + BDJ	CT	CHD	6 months	⑪
Wei, N. 2023 [57]	30	30	52.98 ± 4.97	54.11 ± 4.91	CT + BDJ	CT	AMI	1 month	①③
Wu,M.Z 2024 [58]	51	50	64.27 ± 4.61	64.74 ± 4.57	CT + BDJ	CT	CHD	3 months	①②③⑥
Xiao, D. 2024 [59]	66	66	52.96 ± 3.97	52.84 ± 4.68	CT + BDJ	CT	CHD	12 weeks	①③④⑤
Yan, W. 2020 [60]	32	32	59.25 ± 9.75	55.03 ± 11.18	CT + BDJ	CT	ACS	3 months	②⑧⑩
Yao, H. Y. 2019 [61]	38	38	NA	NA	CT + BDJ	CT	ACS	1 month	①②
Yao,H.Y 2022 [62]	28	28	62.94 ± 1.33	62.51 ± 1.69	CT + BDJ	CT	ACS	3 months	①②⑪
Yu, X. Y. 2022 [63]	50	50	60.02 ± 8.66	61.38 ± 9.21	CT + BDJ	CT	CHD	6 months	①②③
Yu, Y. Y. 2022 [64]	53	53	61.13 ± 11.06	60.4 ± 11.37	CT + BDJ	CT	AMI	6 months	①②③
Zhang, D. 2024 [65]	55	55	61.4 ± 8.5	62.8 ± 7.5	CT + BDJ	CT	AMI	3 months	①②
Zhang, Q. 2024 [66]	45	45	65.8 ± 6.2	64.3 ± 6.0	CT + BDJ	CT	AMI	1 month	①②
Zhang, X. Y. 2023 [67]	60	60	53.8 ± 14.2	52.4 ± 13.6	CT + BDJ	CT	AMI	3 months	①⑪
Zhang, Y. 2021 [68]	40	40	60.48 ± 13.28	65.53 ± 13.23	CT + BDJ	CT	CHD	3 months	①②⑪
Zhang, Z. L. 2019 [69]	33	34	59.42 ± 7.022	58.65 ± 7.027	CT + BDJ	CT	AMI	8 weeks	①⑥⑧⑨⑪
Zhao, K. 2022 [70]	41	41	61.63 ± 5.91	61.89 ± 6.02	CT + BDJ	CT	CHD	3 months	①②
Zheng, H. Y. 2023 [71]	45	45	57.44 + 7.32	56.38 ± 7.31	CT + BDJ	CT	STEMI	3 months	①③⑦⑧
Zhou, S. 2024 [72]	35	35	59.56 ± 5.33	59.60 ± 5.28	CT + BDJ	CT	CHD	3 months	①③
Zhou, W. J. 2021 [73]	30	30	64.63 ± 7.09	66.37 ± 7.19	CT + BDJ	CT	ACS	3 months	④⑪
Zong, S. 2022 [74]	50	50	57.26 ± 6.84	56.91 ± 7.34	CT + BDJ	CT	STEMI	2 weeks	①⑩⑧

ACS, Acute Coronary Syndrome; CHD, Coronary Heart Disease; CT, conventional treatment; STEMI, ST-segment Elevation Myocardial Infarction; SAP, Stable Angina Pectoris; ①LVEF, Left Ventricular Ejection Fraction; ②6MWT, 6-Minute Walk test; ③MACE, Major Adverse Cardiovascular Events; ④SAS, the Self-rating Anxiety Scale; ⑤ SDS, the Self-rating Depression Scale; ⑥NT-proBNP, N-terminal pro-Brain Natriuretic Peptide; ⑦cTnI, cardiac troponin I; ⑧AT, Anaerobic Threshold; ⑨METs, Metabolic Equivalents; ⑩VO_2_peak, Peak Oxygen Uptake; ⑪SAQ, the Seattle Angina Questionnaire

### Quality assessment of studies

**Fig. 2:**
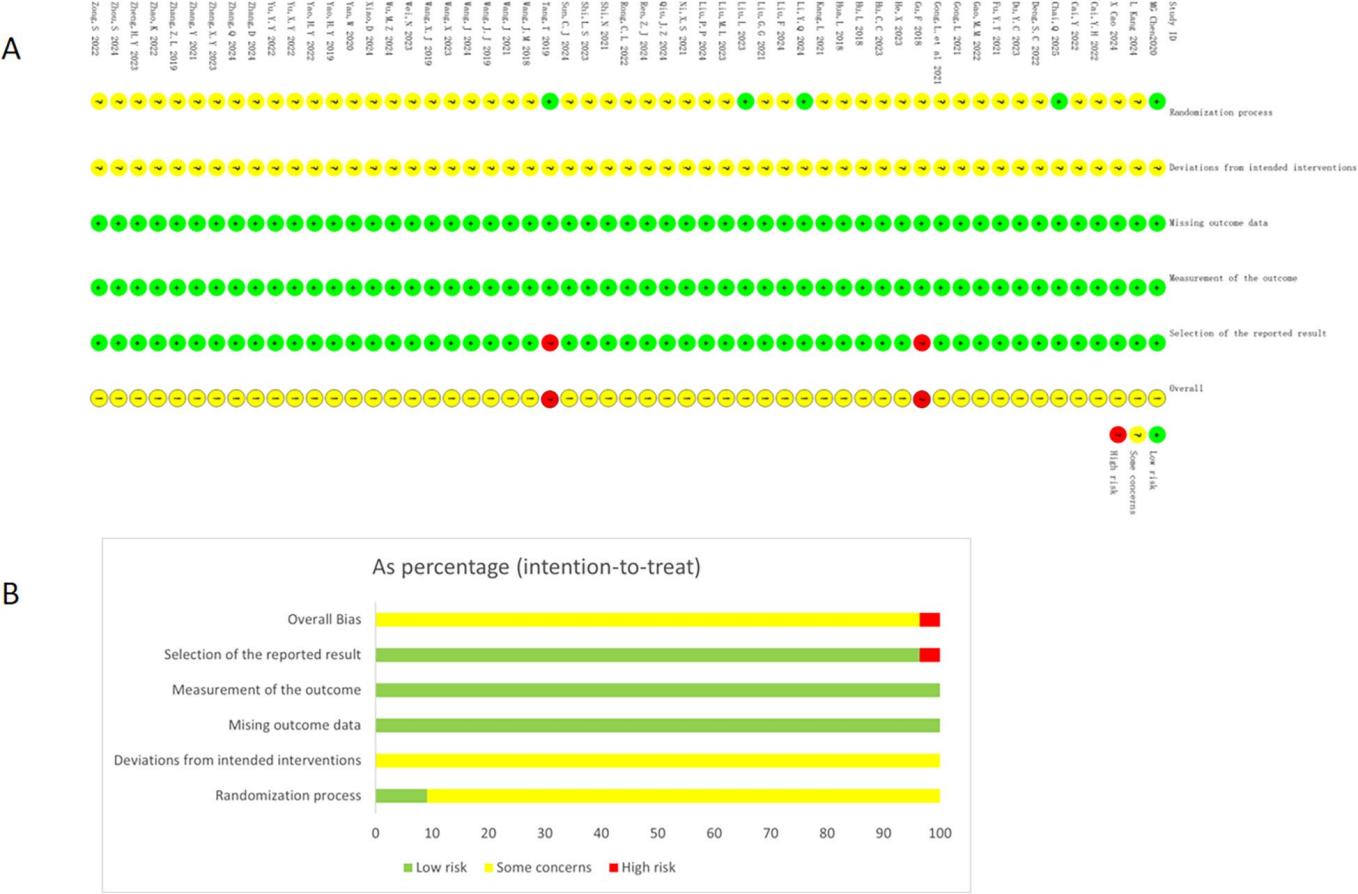
**A** Risk of bias summary; **B** risk of bias graph

A total of 56 papers were included in this study, in which 33 studies [20, 21, 24–27, 32, 34, 36, 37, 39, 42–45, 47–49, 53–55, 59, 63, 65–74] used the random number table, 15 studies [22, 28, 29, 38, 40, 41, 46, 50, 51, 56–58, 60, 61, 64] only mentioned randomization without detailing the randomization scheme, five studies [14, 23, 30, 33, 35], where randomization was performed by computer, and three studies [31, 52, 62] were randomization was performed by lottery. Only five studies [22, 23, 37, 40, 50] reported the use of opaque envelopes to conceal the randomization program. No studies reported the blinding of participants or researchers. The quality assessment of the included RCTs is shown in Fig. 2A, B.

### Meta-analysis of primary outcomes

#### LVEF

**Fig. 3:**
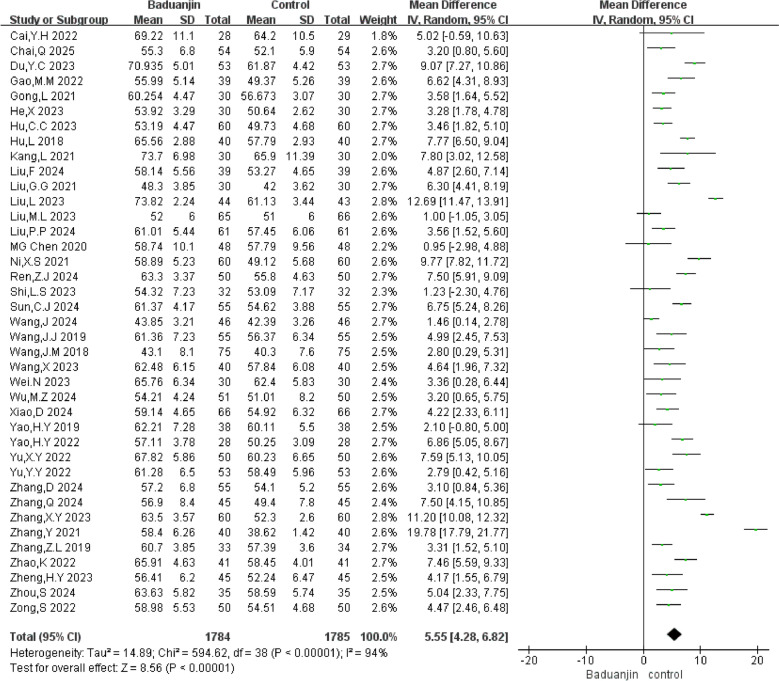
Forest plot of LVEF

A total of 39 RCTs with 3569 participants were analyzed in the forest plot. Compared with the control group, the meta-analysis results showed that Baduanjin exercise could improve LVEF (MD = 5.55%, 95% CI [4.28%, 6.82%], *P* < 0.01, I^2^ = 94%) (Fig. 3).

#### 6MWT

**Fig. 4:**
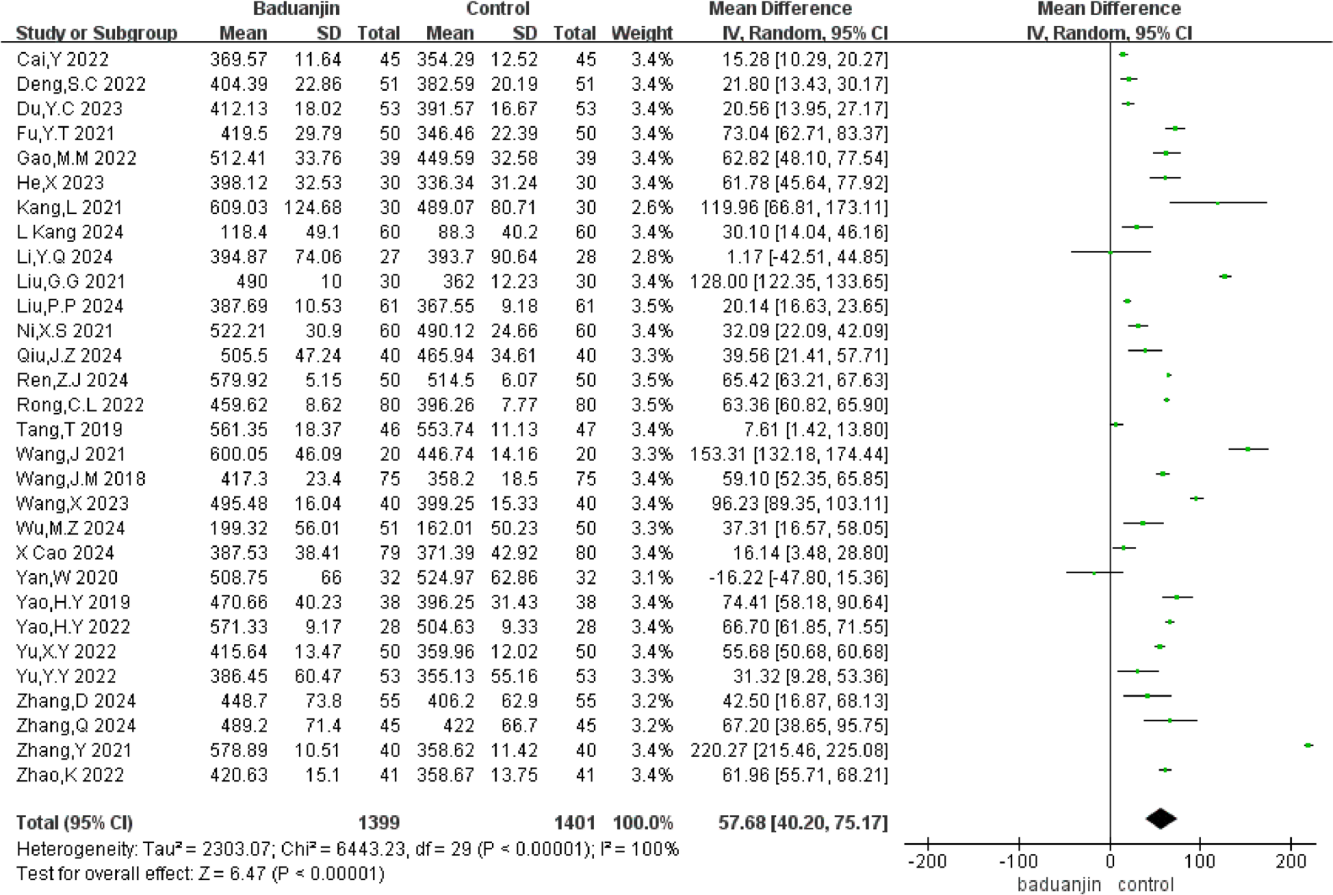
Forest plot of 6MWT

A total of 30 RCTs with 2800 participants were analyzed in the forest plot. Compared with the control group, the meta-analysis results showed that Baduanjin exercise could improve 6MWT (MD = 57.68m, 95% CI [40.20m, 75.17m], *P* < 0.01, *I*^2^ = 100%) (Fig. 4).

### Meta-analysis of secondary outcomes

#### MACE

MACE includes statistics on rehospitalization rates, mortality, recurrent Heart failure, and recurrent angina pectoris. Meta-analysis of 21 RCTs showed that the control group had a higher risk of MACE compared to the Baduanjin group (RR = 0.33, 95% CI [0.26, 0.42], *P* < 0.01, *I*^2^ = 0%) (Table 2).

**Table 2:** Mean difference (95%CIs) of the MACE, SAQ, CPET, Anxiety and Depression Scale, and Biomarkers of cardiac function

Secondary outcomes	Sample size	MD (95%CI)	*P*	I^2^ (%)
MACE	2064	0.33 (0.26,0.42)	< 0.01	0
Rehospitalization rates	918	0.21 (0.13,0.36)	< 0.01	0
Recurrent angina pectoris	1373	0.40 (0.25,0.63)	< 0.01	0
Recurrent heart failure	657	0.30 (0.14,0.63)	< 0.01	0
Mortality	806	0.20 (0.07,0.52)	< 0.01	0
SAQ				
PL	1228	7.49 (4.78, 10.20)	< 0.01	91
AS	1228	12.88 (10.76, 15.00)	< 0.01	77
AF	1228	10.90 (6.05, 15.75)	< 0.01	98
TS	1228	8.04 (2.30, 13.78)	< 0.01	98
DS	1228	11.30 (5.14, 17.45)	< 0.01	98
CPET				
AT	585	1.18 (0.66, 1.69)	< 0.05	59
METs	354	0.61 (0.32, 0.90)	< 0.01	83
VO_2_ peak	651	1.81 (0.82, 2.80)	< 0.01	96
Anxiety and Depression Scale				
SAS	840	− 7.01 (− 8.05, − 5.96)	< 0.01	58
SDS	660	− 6.67 (− 8.34, − 5.00)	< 0.01	89
Biomarkers of cardiac function				
NT-pro BNP	726	− 32.63 (− 63.83, − 1.43)	< 0.01	99
cTNI	448	− 0.13 (− 0.22, 0.05)	< 0.01	84

#### SAQ

A total of 12 studies reported SAQ. The SAQ was divided into five dimensions, including physical limitation (PL), angina stability (AS), angina frequency (AF), treatment satisfaction (TS), and disease knowledge (DS). The meta-analysis results showed that Baduanjin exercise could improve PL (MD = 7.49 points, 95% CI [4.78 points, 10.20 points], *P* < 0.01, *I*^2^ = 91%), AS (MD = 12.88 points, 95% CI [10.76 points, 15.00 points], *P* < 0.01, *I*^2^ = 77%), DS (MD = 11.30 points, 95% CI [5.14 points, 17.45 points], *P* < 0.01, *I*^2^ = 98%), AF (MD = 10.90 points, 95% CI [6.05 points, 15.75 points], *P* < 0.01, *I*^2^ = 98%), and TS (MD = 8.04 points, 95% CI [2.30 points, 13.78 points], *P* < 0.01, *I*^2^ = 98%) compared with the control group (Table 2).

#### VO_2_peak, AT, and METs

Seven studies reported cardiopulmonary exercise test which showed that performing Baduanjin exercise could improve the VO_2_ peak (MD = 1.81 mL/kg/min, 95% CI [0.82 mL/kg/min, 2.80 mL/kg/min], *P* < 0.01, *I*^2^ = 96%) compared to the control groups. Six studies improved AT (MD = 1.18 mL/kg/min, 95% CI [0.66 mL/kg/min, 1.69 mL/kg/min], *P* < 0.01, *I*^2^ = 96%), and Five studies improved the METs (MD = 0.61 METs, 95% CI [0.32 METs, 0.90 METs], *P* < 0.01, *I*^2^ = 83%) (Table 2).

#### SAS and SDS

A meta-analysis of nine studies showed that Baduanjin had a significant effect on reducing SAS score compared with the control group (MD = − 7.01 points, 95% CI [− 8.05 points, − 5.96 points], *P* < 0.01, *I*^2^ = 58%). Seven studies showed that Baduanjin had a significant effect on reducing the SDS score compared to the control group (MD = − 6.67 points, 95% CI [− 8.34 points, − 5.00 points], *P* < 0.01, *I*^2^ = 89%) (Table 2).

### Safety evaluation

Eleven studies [21, 23, 35–37, 41, 45, 52, 55, 62, 67] reported safety assessments. In the Baduanjin group, there were two cases of upper limb pain, one case of vertigo and weakness, one case of sleep disorder, and one case of constipation. In the control group, there was one case of palpitation, four cases of vertigo and weakness, nine cases of sleep disorder, and eight cases of constipation, which could be relieved after resting. The differences were statistically significant, indicating that the Baduanjin exercise did not increase adverse events and is safe.

We also evaluated myocardial injury biomarkers, including NT-proBNP and cTnI, as safety indicators. The results showed that Baduanjin significantly reduced NT-proBNP (MD = − 32.63 pg/mL, 95% CI [− 63.83 pg/mL, − 1.43 pg/mL], P < 0.01, *I*^2^ = 99%) cTNI levels (MD = − 0.13 ng/mL, 95% CI [− 0.22 ng/mL, − 0.05 ng/mL], *P* < 0.01, *I*^2^ = 84%) compared to control group.

### Subgroup analysis

We performed subgroup analysis of the primary outcomes (LVEF and 6MWT) according to the cardiac diagnosis type (ACS vs. CCS), aerobic exercise implementation in control groups (Present/Absent), and intervention duration (≤ 1 month, 1–3 months, ≥ 3 months). Subgroup results are shown in Table 3.

**Table 3:** Results of subgroup analysis of LVEF and 6MWT

Outcome	Stratification	Subgroup	MD (95%CI)	*I*^2^(%)	*P*
LVEF	Cardiac diagnosis	CCS	7.21% (4.85, 9.57)	94.5	< 0.01
		ACS	4.70% (3.20, 6.20)	92.9	< 0.01
	Aerobic exercise in control group	without aerobic exercise	6.46% (4.85, 8.06)	94.8	< 0.01
		with aerobic exercise	3.58% (2.66, 4.51)	46.0	< 0.01
	Intervention duration	≤ 1 month	5.04 (1.03, 9.06)	96.6	< 0.01
		1–3 months	5.76 (4.18, 7.33)	93.6	< 0.01
		> 3 months	5.41 (3.15, 7.66)	84.7	< 0.01
6MWT	Cardiac diagnosis	CCS	66.70m (32.70, 100.70)	99.7	< 0.01
		ACS	49.58m (32.25, 66.92)	99.9	< 0.01
	Aerobic exercise in control group	without aerobic exercise	70.50m (47.78, 93.10)	99.7	< 0.01
		with aerobic exercise	36.28m (21.89, 50.66)	94.3	< 0.01
	Intervention duration	≤ 1 month	45.01m (21.64, 68.38)	98.8	< 0.01
		1–3 months	59.42m (21.33, 97.50)	99.7	< 0.01
		> 3 months	59.88m (46.21,73.54)	96.8	< 0.01

Subgroup analysis showed that performing Baduanjin exercise alone was superior to the combination of the Baduanjin and aerobic exercise, considering the improvement in LVEF (MD = 6.46%, 95% CI 4.85–8.06%) and 6MWT (MD = 70.50 m, 95% CI 47.78–93.10 m). Classified as a low-intensity aerobic exercise, Baduanjin is characterized by slow, gentle movements and low energy expenditure. Its emphasis on deep diaphragmatic breathing reduces heart rate and myocardial oxygen consumption, thereby alleviating cardiac load. The rigorous low-intensity design of Baduanjin (Borg RPE scale 11–14, target Heart rate 50%) serves as the cornerstone of its safety profile [75]. When combined with conventional aerobic exercise, however, the cumulative intensity may exceed safety thresholds, particularly in cardiac patients, leading to a paradoxical surge in myocardial oxygen demand that negates the inherent low-intensity advantages of Baduanjin [76].

In terms of cardiac type, subgroup analysis showed that patients with CCS who received the Baduanjin had higher improvements in LVEF (MD = 7.21%, 95% CI 4.85–9.57%) and 6MWT (MD = 66.70 m, 95% CI 32.70–100.70 m) than those in the ACS group (LVEF: 4.70%, 3.20–6.20; 6MWT: 49.58 m, 32.25–66.92). This discrepancy likely stems from fundamental pathophysiological differences between these clinical entities. ACS is an acute cardiovascular event characterized by myocardial ischemia and significant systemic inflammation [77]. In contrast, patients with CCS have persistent autonomic dysfunction with a chronic low-grade inflammatory state [78, 79]. It was also previously mentioned that the Baduanjin can modulate autonomic and inflammatory responses, which is more compatible with the mechanisms of CCS and may be more advantageous in patients with CCS. However, a larger sample is needed to verify the differences between groups.

In terms of intervention time, subgroup analysis showed that the 1–3-month intervention (MD = 5.76%; 95% CI 4.18–7.33%) was significantly more effective than the ≤ 1-month intervention (MD = 5.04%; 95% CI 1.03–9.06%) and the > 3-month intervention (MD = 5.41%; 95% CI 3.15–7.66%) in terms of LVEF improvement. In terms of 6MWT improvement, the > 3 month intervention (MD = 59.88 m; 95% CI 46.21–73.54 m) was more effective than the ≤ 1 month intervention (MD = 45.01 m; 95% CI 21.64–68.38 m) and the 1–3-month intervention (MD = 59.42 m; 95% CI 21.33–97.50 m). It is suggested that cardiac function can stabilize after approximately one to three months of intervention; however, there is still room for cardiorespiratory endurance to improve over time as it continues to develop, which is consistent with previous studies [80]. The study showed that the most significant improvement in LVEF at three months compared to Nine months. In contrast, the 6MWT relies on the coordination of cardiorespiratory function and muscle strength and is more sensitive to long-term recovery.

### Meta-regression analysis and sensitivity analysis

**Fig. 5:**
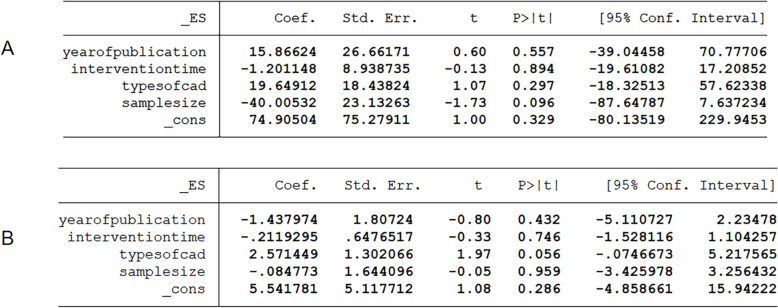
Meta-regression analysis of **A** 6MWT, **B** LVEF

**Fig. 6:**
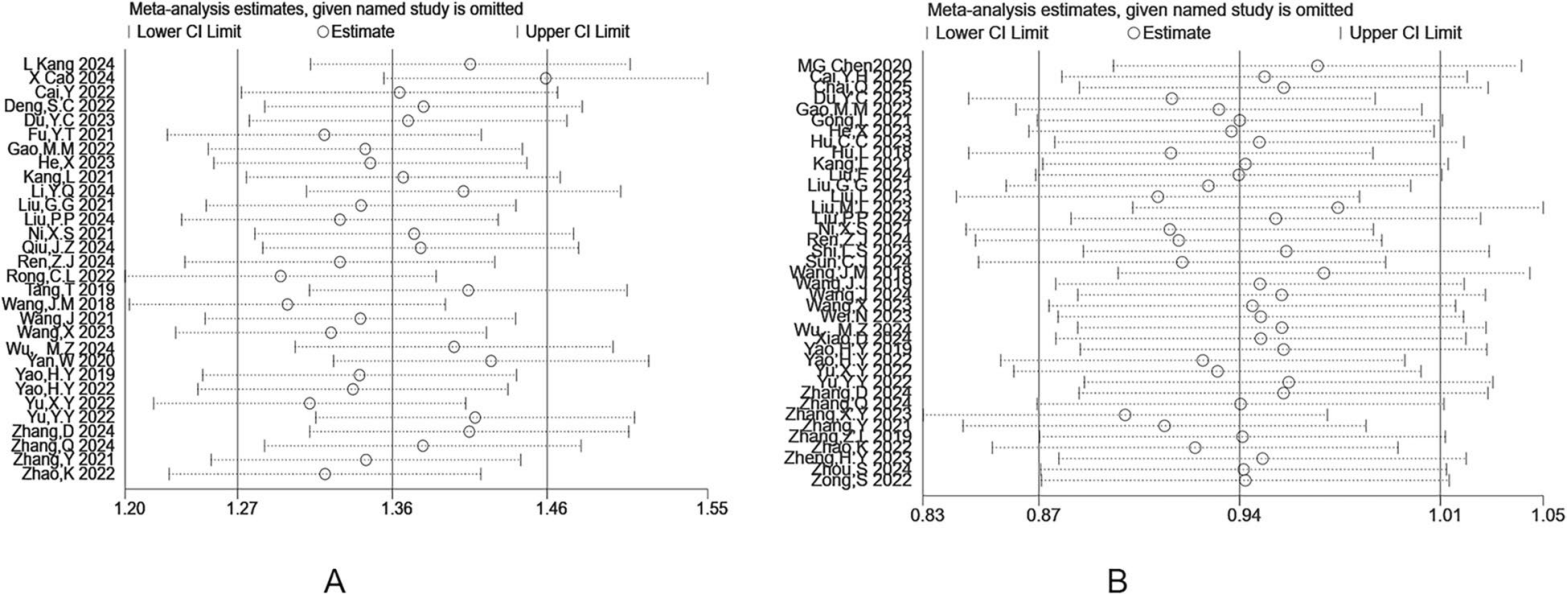
Sensitivity analysis result of **A** 6MWT, **B** LVEF

To further explore sources of heterogeneity, we conducted meta-regression analysis of the Primary outcome. Four characteristics were selected, including (1) year of publication (2018–2020, 2021–2025); (2) duration of intervention (≤ 1, 2, 3 and > 3 months); (3) Types of CHD (CCS or ACS); and (4) sample size (< 60 or ≥ 60). However, meta-regression analysis (Fig. 5) did not find significant effects of the above covariates, suggesting that none of the above characteristics was a source of between-study heterogeneity. Then, we performed a sensitivity analysis, showing that the results were stable (Fig. 6).

### Publication bias

**Fig. 7:**
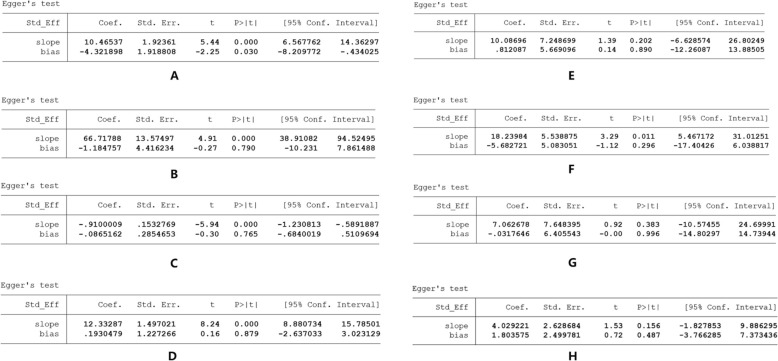
Egger test of **A** LVEF, **B** 6MWT, **C** MACE, **D** SAQ-PL, **E** SAQ-AS, **F** SAQ-DS, **G** SAQ-AF, **H** SAQ-TS

Since more than 10 research reports have reported on LVEF, 6MWT, MACE, and SAQ, we conducted funnel plots and Egger tests. The funnel plots were roughly symmetrical, and the Egger test results yielded *p* values of 0.03, 0.79, 0.765, 0.487, 0.879, 0.89, 0.296, and 0.996, indicating that publication bias was present only for LVEF. We used trim and fill method to fill in four virtual studies, then re-conducted a meta-analysis of all LVEF studies. The results did not reverse, indicating that the combined results were robust (Fig. 7).

### GRADE assessment

The assessment of evidence for inclusion of the outcomes was summarized using the GRADE methodology, as shown in Table 4.

**Table 4:** GRADE assessment for the outcomes

Outcome	Number	Study design	Risk of bias	Inconsistency I2	Indirectness	Imprecision 400	Other considerations	Certainty of evidence
6MWT	30	RCT	Serious	Serious	Not serious	Not serious	None	⨁⨁○○ Low
LVEF	39	RCT	Serious	Serious	Not serious	Not serious	None	⨁⨁○○ Low
MACE	21	RCT	Not Serious	Serious	Not serious	Not serious	None	⨁⨁⨁○ Moderate
SAQ-PL	12	RCT	Serious	Serious	Not serious	Not serious	None	⨁⨁○○ Low
SAQ-AS	12	RCT	Serious	Serious	Not serious	Not serious	None	⨁⨁○○ Low
SAQ-AF	12	RCT	Serious	Serious	Not serious	Not Serious	None	⨁⨁○○ Low
SAQ-TS	12	RCT	Serious	Serious	Not serious	Not serious	None	⨁⨁○○ Low
SAQ-DS	12	RCT	Serious	Serious	Not serious	Not serious	None	⨁⨁○○ Low
AT	6	RCT	Serious	Serious	Not serious	Not Serious	None	⨁⨁○○ Low
METs	5	RCT	Serious	Serious	Not serious	Serious	None	⨁○○○ Very Low
VO_2_ peak	7	RCT	Serious	Serious	Not serious	Not Serious	None	⨁⨁○○ Low
SAS	9	RCT	Serious	Serious	Not serious	Not Serious	None	⨁⨁○○ Low
SDS	7	RCT	Serious	Serious	Not serious	Not Serious	None	⨁⨁○○ Low
NT-proBNP	12	RCT	Serious	Serious	Not serious	Not serious	None	⨁⨁○○ Low
cTNI	5	RCT	Serious	Serious	Not serious	Serious	None	⨁○○○ Very Low

## Discussion

### Summary of main findings

This study comprised a total of 56 RCTs involving 5,152 patients. The results of the meta-analysis showed that the Baduanjin group demonstrated a superior improvement in LVEF, 6MWT, and five dimensions of the SAQ (PL, AS, AF, TS, and DS), as well as NT-proBNP, cTnI, SAS score, and SDS score, compared to the control group. In addition, the Baduanjin group was more effective in reducing the incidence of MACE. The pooled safety data suggested favorable profiles for Baduanjin, with no severe intervention-related incidents reported across the trials. Based on the synthesized evidence from systematic reviews, Baduanjin exercise, as a safe rehabilitative intervention, demonstrates efficacy in enhancing cardiac function recovery, improving exercise tolerance, optimizing quality of life, and ameliorating psychological status in post-PCI patients, thereby highlighting its comprehensive therapeutic value in cardiac rehabilitation.

The therapeutic mechanisms underlying Baduanjin intervention in post-PCI rehabilitation involve multi-system synergistic effects. This practice enhances cardiac electrophysiological stability and reduces arrhythmic risks by restoring autonomic nervous system balance, specifically through increasing heart rate variability and mitigating excessive sympathetic activation [81]. Concurrently, Baduanjin significantly inhibits systemic inflammatory responses, as evidenced by reduced levels of pro-inflammatory factors, such as C-reactive protein and interleukin-6 [82], thereby mitigating vascular endothelial damage and attenuating atherosclerotic progression. In terms of vascular regulation, Baduanjin optimizes coronary microcirculatory perfusion through bidirectional modulation of vasoactive substances, including increased nitric oxide bioavailability and suppressed endothelin-1 secretion [83]. From a biomechanical perspective, its coordinated Movement sequences (e.g., synchronized forearm oscillations and torso rotations) enhance myocardial contractility and stroke volume while reducing cardiac afterload, which alleviates myocardial ischemia collectively [84]. As a low-intensity aerobic exercise, Baduanjin further enhances hemodynamic efficiency and exercise tolerance by augmenting skeletal muscle pump function and cardiorespiratory endurance [85]. These cross-scale physiological adaptations ultimately lead to multiple synergistic effects, effectively improving cardiac function, reducing the incidence of major cardiovascular events, and enhancing postoperative quality of life.

In summary, the results of the subgroup analysis suggest that the Baduanjin intervention should not be combined with other aerobic exercises as much as possible in patients after PCI. CCS patients are a more suitable target population than ACS patients. It is also recommended that the intervention lasts for more than one month. Improvements in LVEF stabilize within one to three months of the intervention, whereas improvements in 6MWT may take longer than three months. Meta-regression and publication bias tests revealed no significant findings, suggesting high stability of the results.

### Strengths compared to other reviews

Three previous studies [86–88] have conducted a meta-analysis of the efficacy of combining Baduanjin exercise with conventional treatment as a control. Specifically, one study [86] included nine articles and focused primarily on the impact of Baduanjin on quality of life through statistical analysis of the SF-36 scale. Another study [88] included 12 articles and focused on the effects on cardiopulmonary function. The third study [87] included 11 articles, mainly investigating the effect of Baduanjin on cardiac function, with LVEF as the primary outcome. In contrast, our study is more comprehensive in terms of Literature retrieval and screening, incorporating a total of 56 articles. We also utilized comprehensive outcome measures to evaluate various aspects. We examined the effects on cardiac function and quality of Life using LVEF, 6MWT, and SAQ. In addition, we assessed the improvement in myocardial injury by looking at NT-proBNP and cTnI levels. We also assessed the relief of depressive and anxious mood using the SAS and SDS scales. Finally, we investigated the effects on cardiopulmonary function by measuring AT, METs, and VO_2_peak. In conclusion, compared to previous studies, our study included a greater number of articles and investigated a broader range of dimensions related to the efficacy of Baduanjin. As a result, our conclusions are more convincing.

This study provides the first empirical evidence regarding the optimal target population for Baduanjin, the feasibility of combining Baduanjin with other aerobic exercises, and its optimal intervention duration. Based on our findings, we recommend: (1) CCS patients as the priority population for Baduanjin interventions; (2) an intervention duration exceeding one month to maximize therapeutic benefits; (3) Baduanjin as a standalone exercise regimen rather than combination therapies. Subgroup analysis indicates that Baduanjin yields cardiac function improvement during the early postoperative phase (≤ 1 month), supporting the feasibility of early intervention. However, > 50% of included studies failed to specify exact initiation timepoints (e.g., 24 h, 72 h, or 7-day post-PCI). Consequently, current evidence cannot determine the optimal intervention window, necessitating prospective trials with stratified timeframes (24h/48h/72h/7d).

Critically, findings are not generalizable to all post-PCI patients. Over 50% of studies exclusively enrolled low-to-moderate-risk cases (Cardiac Rehabilitation Risk Stratification Classes A–B or NYHA Class I–II), explicitly excluding high-risk cohorts (NYHA Class IV or LVEF < 40%). This aligns with the 2020 Chinese Expert Consensus on Exercise Rehabilitation after PCI [89], which permits low-intensity exercise initiation 24 h postoperatively for uncomplicated, low-risk patients. Based on the above, Batanjin exercises are not currently recommended for postoperative PCI in high-risk individuals.

### limitations and perspectives

This study has several limitations that require careful interpretation. First, the methodological quality of the included trials was generally suboptimal, particularly due to inadequate reporting of randomization procedures, allocation concealment, and outcome completeness. While participant blinding in Baduanjin trials remains inherently challenging given the self-administered nature of this intervention, enhanced efforts to maintain investigator and data analyst blinding could have further mitigated potential biases. Second, the predominant reliance on Chinese databases may limit accessibility for international researchers and reduce comparability with Western clinical research frameworks, necessitating cautious interpretation of these findings as hypothesis-generating rather than practice-altering. Third, cardiac biomarker measurements (LVEF, NT-proBNP, cTnI) obtained within 7-day post-revascularization should be interpreted with caution as physiological fluctuations from myocardial stunning, hibernating myocardium, or ischemia–reperfusion injury may transiently compromise their diagnostic reliability. Finally, long-term follow-up data (≥ 12 months), constraining comprehensive safety assessments and sustainability evaluations of Baduanjin's therapeutic effects.

To address these limitations, future research should prioritize multicenter, international RCTs employing double-blind designs and standardized protocols aligned with the Consolidated Standards of Reporting Trials (CONSORT) guidelines. Such trials must incorporate stratified randomization to account for variations in cultural and rehabilitation protocols across healthcare systems, thereby enhancing the generalizability of findings regarding Baduanjin’s efficacy in post-PCI cardiac rehabilitation. Extended follow-up periods with serial biomarker assessments—including a dedicated 7-day post-procedural timepoint—are essential to distinguish between acute procedural effects and sustained therapeutic benefits. Concurrently, systematic documentation of adverse events and mechanistic investigations using advanced imaging modalities (e.g., cardiac MRI perfusion analysis) should be integrated to elucidate the impacts of Baduanjin on myocardial microcirculation and metabolic regulation. These efforts will establish a robust evidence base to evaluate both the long-term prognostic implications and quality-of-life outcomes associated with this traditional exercise regimen in modern cardiology practice.

## Conclusions

Baduanjin can improve cardiopulmonary function, alleviate clinical symptoms, enhance the quality of life, stabilize the mental state, and reduce the incidence of MACE in patients after PCI. However, the quality of the currently included studies is generally low. More rigorous, standardized, and high-quality RCTs are needed to confirm further the efficacy and safety of combining Baduanjin with conventional therapy and to inform clinical practice more effectively.

## Supplementary Information

Additional file 1.

## Data Availability

No datasets were generated or analysed during the current study.

## Abbreviations

CR: Cardiac rehabilitation
CHD: Coronary heart disease
PCI: Percutaneous coronary intervention
ACS: Acute Coronary Syndrome
MACE: Major adverse cardiovascular events
LVEF: Left ventricular ejection fraction
6MWT: 6-Minute walk test
SAS: Self-Rating Anxiety Scale
SDS: Self-Rating Depression Scale
NT-proBNP: N-terminal pro-Brain Natriuretic Peptide
cTnI: Cardiac troponin I
AT: Anaerobic threshold
METs: Metabolic equivalents
VO_2_ peak: Peak oxygen uptake
SAQ: The Seattle Angina Questionnaire
