# Supplementary material for: Effects of Baduanjin exercise on cardiac rehabilitation after percutaneous coronary intervention: a systematic review and meta-analysis of randomized controlled trials
Source: Eur J Med Res. 2025 Sep 29;30:889. doi: 10.1186/s40001-025-03031-2 (PMC12482649; doi:10.1186/s40001-025-03031-2)
Supplement: Supplementary file 1 — Additional file 1. [file 40001_2025_3031_MOESM1_ESM.docx]

Appedix A1: The search strategies of Baduanjin exercise on patients with Percutaneous Coronary Intervention

| **Databases** | **Search strategies** | **Hit Counts** |
| --- | --- | --- |
| **Pubmed**  **(https://pubmed.ncbi.nlm.nih.gov/advanced/)** | #1 "Percutaneous Coronary Intervention"[MeSH Terms]  #2 "coronary intervention percutaneous"[Title/Abstract] OR "coronary interventions percutaneous"[Title/Abstract] OR "intervention percutaneous coronary"[Title/Abstract] OR "interventions percutaneous coronary"[Title/Abstract] OR "percutaneous coronary interventions"[Title/Abstract] OR "percutaneous coronary revascularization"[Title/Abstract] OR "coronary revascularization percutaneous"[Title/Abstract] OR "coronary revascularizations percutaneous"[Title/Abstract] OR "percutaneous coronary revascularizations"[Title/Abstract] OR "revascularization percutaneous coronary"[Title/Abstract] OR "revascularizations percutaneous coronary"[Title/Abstract]  #3 #1 AND #2  #4 Baduanjin  #5 #3 AND #4 | 8 |
| **Web of science**  **(https://www.webofscience.com/wos/alldb/advanced-search)** | #1 TS=(Baduanjin)  #2 Percutaneous Coronary Intervention OR Coronary Intervention, Percutaneous OR Coronary Interventions, Percutaneous OR Intervention, Percutaneous Coronary OR Interventions, Percutaneous Coronary OR Percutaneous Coronary Interventions OR Percutaneous Coronary Revascularization OR Coronary Revascularization, Percutaneous OR Coronary Revascularizations, Percutaneous OR Percutaneous Coronary Revascularizations OR Revascularization, Percutaneous Coronary OR Revascularizations, Percutaneous Coronary  #3 #1 AND #2 | 7 |
| **Embase**  **(https://www.embase.com)** | #1 'baduanjin'/exp OR baduanjin  #2 Percutaneous Coronary Intervention OR Coronary Intervention, Percutaneous OR Coronary Interventions, Percutaneous OR Intervention, Percutaneous Coronary OR Interventions, Percutaneous Coronary OR Percutaneous Coronary Interventions OR Percutaneous Coronary Revascularization OR Coronary Revascularization, Percutaneous OR Coronary Revascularizations, Percutaneous OR Percutaneous Coronary Revascularizations OR Revascularization, Percutaneous Coronary OR Revascularizations, Percutaneous Coronary  #3 #1 AND #2 | 2 |
| **The Cochrane Library**  **(https://www.cochranelibrary.com/advanced-search)** | #1 Baduanjin  #2 Percutaneous Coronary Intervention OR Coronary Intervention, Percutaneous OR Coronary Interventions, Percutaneous OR Intervention, Percutaneous Coronary OR Interventions, Percutaneous Coronary OR Percutaneous Coronary Interventions OR Percutaneous Coronary Revascularization OR Coronary Revascularization, Percutaneous OR Coronary Revascularizations, Percutaneous OR Percutaneous Coronary Revascularizations OR Revascularization, Percutaneous Coronary OR Revascularizations, Percutaneous Coronary  #3 #1 AND #2 | 7 |
| **CNKI**  **(https://www.cnki.net/)** | (主题:八段锦AND(主题:经皮冠状动脉介入 OR 冠状动脉介入 OR PCI OR 介入术 OR 支架植入术 OR 支架置入术 OR 球囊血管成形术) | 136 |
| **WanFang**  **(https://www.wanfangdata.com.cn/)** | 题名或关键词:(八段锦) AND (题名或关键词:经皮冠状动脉介入 OR 冠状动脉介入 OR PCI OR 介入术 OR 支架植入术 OR 支架置入术 OR 球囊血管成形术） | 105 |
| **VIP**  **(http://qikan.cqvip.com/index.html)** | M=八段锦 AND M=(经皮冠状动脉介入 OR 冠状动脉介入 OR PCI OR 介入术 OR 支架植入术 OR 支架置入术 OR 球囊血管成形术) | 74 |
| **SinoMed**  **(http://www.sinomed.ac.cn/index.jsp)** | "八段锦"[标题:智能] AND "经皮冠状动脉介入 OR 冠状动脉介入 OR PCI OR 介入术 OR 支架植入术 OR 支架置入术 OR 球囊血管成形术"[标题:智能] | 76 |
| **the Chinese Clinical Trial Registry (http://www.chictr.org.cn/index.aspx)** | 在“注册题目”检索框中，输入“八段锦” | 161 |
| **ClinicalTrials.gov (www.clinicaltrials.gov/)** | In the search box for "Intervention/treatment", type " Baduanjin"； | 3 |
